# Developmental Function of the PHR Protein RPM-1 Is Required for Learning in *Caenorhabditis elegans*

**DOI:** 10.1534/g3.115.021410

**Published:** 2015-10-09

**Authors:** Andrew C. Giles, Karla J. Opperman, Catharine H. Rankin, Brock Grill

**Affiliations:** * Department of Neuroscience, The Scripps Research Institute, Scripps Florida, Jupiter, Florida 33458; † Department of Psychology, University of British Columbia, Vancouver, British Columbia V6T 1Z4, Canada; ‡ Brain Research Centre, University of British Columbia, Vancouver, British Columbia V6T 2B5, Canada

**Keywords:** *C. elegans*, PHR protein, RPM-1, habituation, learning

## Abstract

The PAM/Highwire/RPM-1 (PHR) proteins are signaling hubs that function as important regulators of neural development. Loss of function in *Caenorhabditis elegans rpm-1* and *Drosophila Highwire* results in failed axon termination, inappropriate axon targeting, and abnormal synapse formation. Despite broad expression in the nervous system and relatively dramatic defects in synapse formation and axon development, very mild abnormalities in behavior have been found in animals lacking PHR protein function. Therefore, we hypothesized that large defects in behavior might only be detected in scenarios in which evoked, prolonged circuit function is required, or in which behavioral plasticity occurs. Using quantitative approaches in *C. elegans*, we found that *rpm-1* loss-of-function mutants have relatively mild abnormalities in exploratory locomotion, but have large defects in evoked responses to harsh touch and learning associated with tap habituation. We explored the nature of the severe habituation defects in *rpm-1* mutants further. To address what part of the habituation circuit was impaired in *rpm-1* mutants, we performed rescue analysis with promoters for different neurons. Our findings indicate that RPM-1 function in the mechanosensory neurons affects habituation. Transgenic expression of RPM-1 in adult animals failed to rescue habituation defects, consistent with developmental defects in *rpm-1* mutants resulting in impaired habituation. Genetic analysis showed that other regulators of neuronal development that function in the *rpm-1* pathway (including *glo-4*, *fsn-1*, and *dlk-1*) also affected habituation. Overall, our findings suggest that developmental defects in *rpm-1* mutants manifest most prominently in behaviors that require protracted or plastic circuit function, such as learning.

The PHR proteins, named for the orthologs PAM (MYCBP2) in *Homo sapiens*, Highwire in *Drosophila melanogaster*, and RPM-1 in *Caenorhabditis elegans*, are important regulators of neuronal development (reviewed in Po *et al.* 2010). PHR proteins function as intracellular signaling hubs that regulate the activity of multiple downstream signaling pathways that include: MAP kinases (Baker *et al.* 2015; Collins *et al.* 2006; Lewcock *et al.* 2007; Nakata *et al.* 2005; Nix *et al.* 2011; Yan *et al.* 2009), the PP2C phosphatase PPM-2 that inhibits the DLK-1 MAP3K in *C. elegans* (Baker *et al.* 2014), the tuberous sclerosis complex (Han *et al.* 2012; Murthy *et al.* 2004), the microtubule binding protein RAE-1 (Grill *et al.* 2012; Tian *et al.* 2011), Rab signaling (Grill *et al.* 2007), and beta-catenin signaling (Tulgren *et al.* 2014). In *C. elegans*, *rpm-1* loss-of-function (lf) mutants have strong defects in neuronal development. The mechanosensory neurons that mediate gentle touch responses have axon termination defects (Schaefer *et al.* 2000), and sensitizing genetic backgrounds have been used to reveal abnormalities in axon guidance (Li *et al.* 2008). These neurons also have defects in synapse formation with downstream command interneurons (Schaefer *et al.* 2000). Similar defects in axon termination and synaptogenesis are found in motor neurons (Opperman and Grill 2014; Zhen *et al.* 2000). Additionally, the interneurons of *rpm-1* mutants have postsynaptic defects in which glutamate receptor trafficking is abnormal (Park *et al.* 2009).

Despite strong developmental abnormalities, very few behavioral phenotypes caused by loss of PHR protein function have been identified in any organism. In adult *Drosophila Highwire* mutants, mild walking defects have been qualitatively reported (Wan *et al.* 2000), and altered long-term memory occurs in aversive olfactory conditioning (Huang *et al.* 2012). In *C. elegans*, *rpm-1* (lf) mutants are mildly hypersensitive to paralysis induced by the acetylcholinesterase inhibitor aldicarb (Vashlishan *et al.* 2008). Although *rpm-1* mutants are reported to move normally, *rpm-1* (lf) has a synthetic synaptic phenotype with *syd-2* (lf) mutants that causes impaired locomotion (Liao *et al.* 2004; Noma *et al.* 2014). The behavioral consequences of impairing Phr1 (the mouse PHR ortholog) have not been tested because Phr1 null mice die shortly after birth, presumably from reduced diaphragm innervation and impaired breathing (Bloom *et al.* 2007; Burgess *et al.* 2004; Lewcock *et al.* 2007).

Understanding how developmental abnormalities caused by loss of PHR protein function impact behavior is critical, if we are to achieve an overall understanding of how PHR proteins function in the nervous system. For more than a decade, we have known that RPM-1 is broadly expressed in the *C. elegans* nervous system and that *rpm-1* (lf) mutants have pronounced defects in neuronal development (Abrams *et al.* 2008; Schaefer *et al.* 2000; Zhen *et al.* 2000). And yet, relatively few behavioral abnormalities have been reported in *rpm-1* mutants. Previous studies have assessed spontaneous or baseline behaviors. Thus, we hypothesized that RPM-1 dependent development might be important for behaviors mediated by evoked, protracted, or plastic neuronal function. This prompted us to analyze *rpm-1* mutants for three quantifiable *C. elegans* behaviors that are mediated by neurons that express RPM-1: 1) exploratory locomotion, a spontaneous behavior (Gray *et al.* 2005; Hills *et al.* 2004; Pierce-Shimomura *et al.* 1999; Zhao *et al.* 2003); 2) harsh touch response, a strongly evoked behavior (Chatzigeorgiou *et al.* 2010; Li *et al.* 2011; Way and Chalfie 1989); and 3) tap habituation, a form of short-term learning that results in a decreased response after repeated tap stimulation (Giles and Rankin 2009; Rankin *et al.* 1990). Although we observed only mild defects in exploratory locomotion in *rpm-1* (lf) mutants, defects were much stronger for the harsh touch response and tap habituation. Given the severity of habituation defects in *rpm-1* mutants, we explored this phenotype in more detail. We analyzed the role of RPM-1 in different cells of the tap habituation circuit and found that RPM-1 affects habituation by functioning in the mechanosensory neurons. Impaired habituation in *rpm-1* mutants was not rescued by expression of RPM-1 in adulthood, which suggests that habituation defects are a developmental consequence of *rpm-1* dysfunction. Overall, our findings suggest that loss of *rpm-1* function has wide-ranging consequences on behavior, in particular plastic behavior such as short-term learning.

## Materials and Methods

### Genetics

*C. elegans* strains/alleles were received from the *Caenorhabditis* Genetics Center (unless otherwise specified) and maintained by the use of standard protocols (Brenner 1974; Stiernagle 2006). N2 was used as the wild-type reference strain. Mutant alleles were genotyped by polymerase chain reaction and analyzed by product size or digestion with restriction endonucleases. Genotyping was described previously for all alleles with the exception of the following alleles: 1) *rpm-1* (*ok364*) used forward = GAGTTGGGCTGTATGGAGGA, reverse = GACGGAATTTCGTTGGAGAA, internal = TGGAATGGATTCTGGTGGAT primers for wild-type amplicon = 1590 bp and mutant = 753 bp; 2) *pha-1* (*e2123*) used forward = CAATCGACTGGAGCTTCGTG, and reverse = GTTGTCGCGCACTACTGAATC primers and was digested with *Acc*I restriction endonuclease (New England BioLabs) for wild-type product = 934 bp and mutant = 427 bp and 507 bp. Other alleles included: *rpm-1* (*ju44*), *dlk-1* (*tm4024*), *glo-4* (*ok623*), and *fsn-1* (*hp1*).

### Transgenics

Transgenic animals were created by injecting mixtures of purified plasmid DNA into the gonad of young adult hermaphrodites (Mello *et al.* 1991). Injected worms carried the *pha-1* temperature-sensitive, lethal mutant allele (*e2123*) and the injection mixture contained pBX (wild-type *pha-1* locus; 20 ng/μL). This allowed positive selection of transgenic progeny using a restrictive temperature (Granato *et al.* 1994). PHA-1 is only needed early in development, so positive selection was performed at 23° for the first 16 hr after egg-lay. A plasmid carrying P_myo-2_mCherry (1 ng/μL), which induces red fluorescence in the pharynx, also was included in injection mixtures to visually confirm positive transgenic animals. We used the following plasmids: pCZ160 (P_rpm-1_RPM-1), a gift from Dr. Yishi Jin (Zhen *et al.* 2000); pSAM13 (P_mec-3_RPM-1), a gift from Dr. Michael Nonet (Schaefer *et al.* 2000); pOR822 (P_glr-1_RPM-1), a gift from Dr. Christopher Rongo (Park *et al.* 2009); pCFJ90 (P_myo-2_mCherry) from Addgene; pBG-GY497 (P_mec-3_GFP) and pBG-137 (P_unc-25_RPM-1) that were previously described (Opperman and Grill 2014). We constructed the following plasmids by using standard techniques including polymerase chain reaction, restriction endonuclease digestion (New England BioLabs), T4 ligase cloning (New England BioLabs), and TOPO-TA and Gateway cloning (Life Technologies): pBG-223 (P_unc-129_RPM-1) that had a promoter consisting of 4821 bp of genomic DNA upstream of the *unc-129* start codon and the *rpm-1* coding region and 3′ UTR from pSAM13, pBG-208 (P_hsp-16.2_RPM-1::GFP) that had a promoter consisting of 392 bp of genomic DNA upstream of the *hsp-16.2* start codon and the RPM-1::GFP and 3′ UTR sequence from pBG-46 (Opperman and Grill 2014), and pBG-GY581 (P_hsp-16.2_GFP) that had the same promoter as pBG-208 and the same GFP coding sequence and *unc-54* 3′ UTR as pBG-GY497. Plasmids for RPM-1 expression were included in injection mixtures at 10−40 ng/μL, whereas GFP containing plasmids were included at 3−12 ng/μL to ensure comparable molar ratios of plasmids were injected. pBluescript was added to achieve a total DNA concentration of 100 ng/μL in each injection mixture. DNA concentrations were estimated based on fragment intensity after plasmid digestion, gel electrophoresis, and imaging using ethidium bromide. A 1-kb ladder (New England Biolabs) was used as a standard for quantitation.

### Behavioral recording

Behavioral recordings were taken using a Multi-Worm Tracker (Swierczek *et al.* 2011) with a 12 Mpixel CMOS sensor camera-link camera (CSC12M25BMP19-01B; Toshiba-Teli), a lens adaptor (F-TAR2), a Rodagon-F 50-mm f/2.8 lens (0703-089-000-24; Qioptiq), and a PCIe-1433 camera-link frame grabber (781169-01; National Instruments). The camera was mounted at a distance above the assay plate so that pixel size was 23.3 µm. A region of interest was set to exclude the area close to the edge of the assay plate where conditions for tracking animals were poor. The frame rate of recordings was ∼13.5 Hz.

### Exploratory locomotion

Assay plates were 60 × 15-mm Petri dishes (VWR, 25384-090) filled with nematode growth medium, but without bacteria. *C. elegans* avoid glycerol, so assay plates were painted with a ring of glycerol around the edge of the agar with a cotton-tipped applicator immediately prior to transferring worms onto the plate. This kept animals from migrating to locations where tracking conditions were poor.

*C. elegans* were cultivated at 23° and were aged 65−85 hr after egg-lay. Thirty animals were transferred from well-fed plates to assay plates by gently picking them with a platinum wire (without the use of bacteria for adhesion of worms). During the transfer, animals were briefly placed on plain agar and allowed to crawl for a few seconds to remove the majority of bacteria from their body. Transfer of 30 animals typically took 5 min, and then the behavior of all animals on the plate was immediately recorded for the following 5 min by use of the Multi-Worm Tracker (MWT).

Choreography (part of the MWT software package) was used to collect centroid positions for each frame during the final 60 sec of the 5-minute recording. The first 4 min were ignored to avoid potential effects caused by incidental stimulation during transfer and placement into the MWT. Objects that were recorded for less than 10 sec were ignored to avoid imaging artifacts. The “Reoutline” and “Respine” plugins were used in combination with the “segment” argument and the “bias” output argument to identify periods of forward and backward locomotion. Change in position between frames was calculated and then a worm’s average speed was calculated by dividing the sum of changes by the total duration across those frames. The mean of animals on a plate was weighted by the proportion of time an animal was recorded moving in a given direction during the 60-sec measurement period. These weighted means were considered single datum and multiple plates were tested per genotype (6−8). Student’s unpaired *t*-tests were used for statistical comparisons and reported in the results and figure legends. Statistical analysis was also verified with Mann-Whitney *U*-tests, which gave comparable findings regarding significance.

### Harsh touch response

Harsh touch was assessed with plates similar to those used for exploratory locomotion, but plates were freshly seeded with a thin bacterial lawn. A total of 10−15 L4 animals (cultivated at 23°) were transferred to each assay plate and cultivated at 23° for 12−24 hr. To test harsh touch response, plates were carefully placed under a dissecting microscope and animals that were not near the edge of the lawn and were moving forward were touched with a platinum wire on their anterior body, just behind the pharynx. The number of body bends that the animal moved backward after the stimulus was recorded (rounded to the closest whole number). Twenty animals were tested per day, and the mean response was calculated and considered a single datum. Multiple days of experiments were tested per genotype (4−6). Student’s unpaired *t*-tests were used for statistical comparisons and reported in the results and figure legends. Statistical analysis was also verified with Mann-Whitney *U* tests, which gave comparable findings regarding significance.

### Tap habituation

The tap stimulus was delivered using a computer controlled linear solenoid as described previously (Swierczek *et al.* 2011; Timbers *et al.* 2013), except with a few modifications. We used a different timer/counter card and connection block (National Instruments, PCI-6601 and CB-68LP) and a custom LabView program was written to control the solenoid using these new components.

Habituation experiments were performed similar to previous studies (Swierczek *et al.* 2011; Timbers *et al.* 2013). Assay plates were similar to those used for exploratory locomotion except 50 µL of OP50 *E. coli* cultured in Lysogeny Broth (LB)-Miller medium was spread onto the plate the day before use. Five gravid adults were placed on the assay plates for 3 hr to lay age-synchronized eggs (∼100). More parents were used for mutant strains or transgenic lines that had a lower egg-laying rate or survival rate. The adults were removed and the plates (with eggs) were cultivated at 20° for 86−96 hr. For experiments that included transgenic animals, all groups were cultivated at 23° for the first 16 hr for *pha-1*−positive selection of transgenes. Then, 30 min before testing, plates were removed from the incubator to acclimatize to room temperature (23°). Plates were secured in the MWT with a lid on the Petri plate. Animals were recorded for 400 sec. Tap stimuli began after the first 100 sec and continued 30 times at a 10-sec interstimulus interval. In some experiments (noted in the figures), 45 stimuli were presented, in which case animals were recorded for 550 sec.

Analysis of the tap response was done using Choreography (MWT analysis software) with custom written scripts (Octave). The “Reoutline” and “Respine” plugins were used in combination with the “segment” argument and the “bias” output argument to process the paths of each animal and the “MeasureReversal” plugin was used to detect reversals within 2 sec of tap stimuli. The probability of reversal in response to tap was estimated by measuring the proportion of animals that reversed within 2 sec of each stimulus. Best-fit exponential curves were fit to the probability *vs.* stimulus data and the habituation level phenotype was measured by estimating the asymptote as the value on the curve at the final stimulus. This value was calculated for each plate as a single datum and multiple plates were tested per genotype/condition (n = 3−6). We used Student’s *t*-tests to compare habituation levels between groups and reported these in the results and figure legends. When possible, we also evaluated comparisons with Mann−Whitney *U*-tests and found comparable significance results. Reported *P*-values were not corrected for multiple comparisons; however, no more than 5 comparisons were conducted for any group and most significant differences were *P* < 0.01, so Bonferroni corrections for five comparisons would still be significant at alpha = 0.05. For the few exceptions, we replicated the experiment but did not always show the data (*e.g.*, habituation of *glo-4 vs. rpm-1* in Figure 6B was replicated but the replication is not shown).

### Heatshock

Stable transgenic lines were selected initially using *pha-1*−positive selection. Once lines were isolated, they were maintained at 15° using the visual P_myo-2_mCherry marker so that temperature shifts could be used to control the heat shock promoter transgenes. For adult-specific expression experiments, 200 transgene-positive young adult animals (∼96 hr after egg-lay when cultivated at 15°) were transferred to an assay plate. The plate was placed in a 33° incubator for 2 hr and then moved to room temperature (23°) for 3 hr to recover. Animals were transferred from the heat shock plate to another assay plate (30 per plate, five plates total), allowed to recover for another hour and then tested. For the intense heat shock protocol, the animals received two sessions of 2-hour heat shock with a 30-min rest at 23° in between and animals were tested for habituation 18−24 hr later. For sustained expression throughout development, experiments were conducted as normal except the cultivation temperature was 23° for the entire time and animals were tested at 72 hr after egg-lay to account for the faster development and aging when cultivated at 23° (Byerly *et al.* 1976). There was no need to visually select transgene-positive animals when animals were grown at 23° because *pha-1* selection occurred.

### Imaging

For analysis of RPM-1::GFP, live adult animals were anesthetized using Levamisole in M9 buffer and visualized using an epifluorescent microscope (Leica CFR5000) with a 40× magnification oil-immersion lens. Images were acquired using a CCD camera (Leica DFC345 FX). Ten animals per strain were observed across two independent days with representative images shown in Figure 5.

### Data availability

All experiments should be repeatable using the information described, links to public databases and cited literature. The following data and materials are available upon request: any strains not available from the *Caenorhabditis* Genetics Center, genotyping reaction details, plasmids.

## Results

### *rpm-1* mutants have defects in exploratory locomotion

RPM-1 is broadly expressed in the nervous system (Schaefer *et al.* 2000; Zhen *et al.* 2000). With regard to the locomotion circuit, RPM-1 is expressed in the motor neurons that control body wall muscles (Zhen *et al.* 2000) and the glutamate-receptor-containing interneurons (Park *et al.* 2009) that coordinate these motor neurons (Chalfie *et al.* 1985). Previous work showed that *rpm-1* (lf) mutants have disorganized synapses in motor neurons, but gross observation of behavior suggested that locomotion was not impaired in these animals (Nakata *et al.* 2005; Schaefer *et al.* 2000; Zhen *et al.* 2000). Thus, although major deficits in locomotion are not present in *rpm-1* mutants, we hypothesized that synapse formation defects in these animals might lead to subtle effects on locomotion.

**Figure 1:**
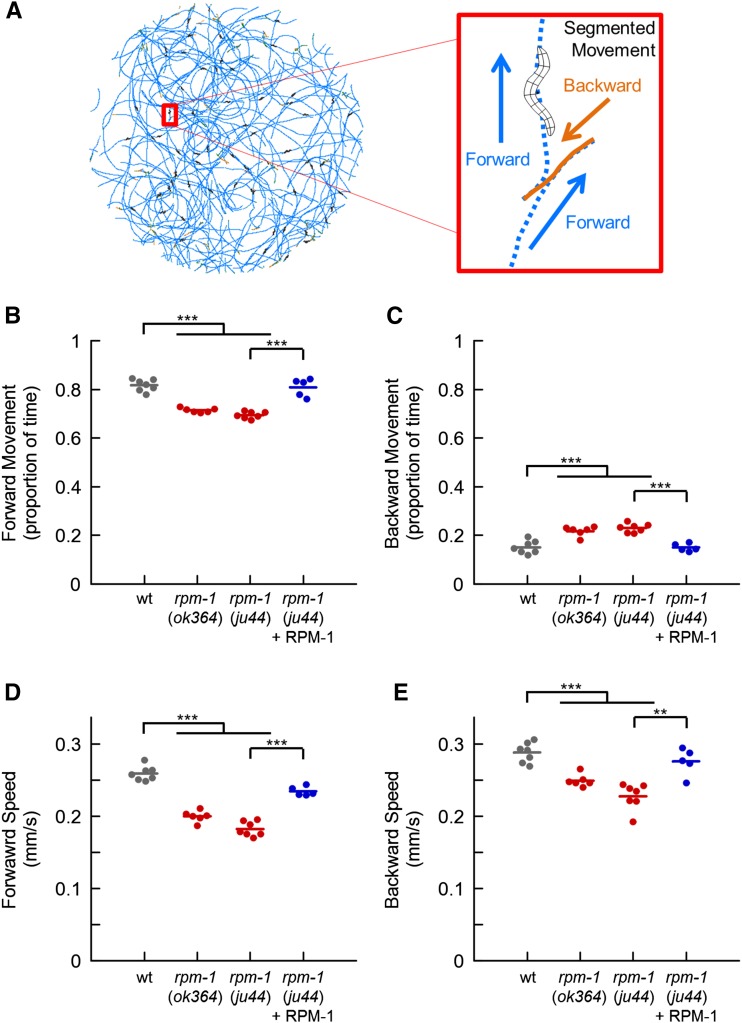
Loss of RPM-1 affects exploratory locomotion. (A) Multi-Worm Tracker was used to quantitatively analyze the path of 30 wild-type *C. elegans* during 5 min of exploratory behavior on an agar plate with no food (left); paths were segmented into forward and backward movement (right). The majority of locomotion is forward, with relatively infrequent backward movement. (B−C) Proportion of time animals spent moving forward (B) and backward (C) during the final minute of observation. Shown are wild-type animals (wt; gray), *rpm-1* (lf) mutants (red), and *rpm-1* mutants with a transgene that uses the native *rpm-1* promoter to express RPM-1 (blue). (D−E) Average speed during the final minute of observation of forward (D) and backward (E) movement for wild-type animals (wt; gray), *rpm-1* mutants (red), and *rpm-1* mutants with a transgene that uses the native *rpm-1* promoter to express RPM-1 (blue). Points represent the mean of 30 animals for a single independent trial. Lines represent the mean of the points for each genotype. For transgenic rescue (blue), each point represents an independently derived transgenic line. **P < 0.01; ***P < 0.001 for Student’s unpaired two-tailed *t*-test between indicated groups.

Exploratory locomotion is well characterized in *C. elegans* (Gray *et al.* 2005; Hills *et al.* 2004; Pierce-Shimomura *et al.* 1999; Zhao *et al.* 2003). When food is not present, animals are forced to actively explore their environment as opposed to foraging locally. During exploration, predominant forward movement is interspersed with reorientation events known as reversals. Using the MWT (Swierczek *et al.* 2011), we simultaneously recorded the locomotion of 30 animals on agar plates without food. Animals were analyzed 5−10 min after removal from food, so we were observing their behavior during the local search phase of exploration (Gray *et al.* 2005). We segmented the animals’ paths into forward and backward movement (Figure 1A). A small portion of locomotion (approximately 5%) was ignored because it could not be characterized as either forward or backward. This included periods when animals were stationary or when animals performed rapid sequential direction changes with large-angle turns that the MWT analysis could not segment properly. As expected, wild-type animals spent most of their time moving forward with small amounts of backward movement during reversals (Figure 1, B and C). We tested two loss-of-function mutants of *rpm-1* for defects in locomotion. *ok364* has a 2221 bp deletion that causes a frameshift a third of the way through the coding sequence (Park *et al.* 2009), and *ju44* has a missense mutation at a conserved cysteine (Zhen *et al.* 2000). These alleles are thought to be nulls. Both *rpm-1* mutants spent significantly less time moving forward compared with wild-type animals (Figure 1B). The reduction in time spent moving forward was reflected by increased time spent moving backward (Figure 1C), as opposed to staying stationary or performing compound/complex reorientations (data not shown). Analysis of the speed of locomotion showed that *rpm-1* mutants moved 15–20% more slowly than wild-type animals during both forward and backward movement (Figure 1, D and E). *rpm-1* mutants that were engineered with transgenic extrachromosomal arrays, in which the native *rpm-1* promoter was used to drive RPM-1 expression, rescued the change in the proportion of time spent moving forward and backward, as well as the defect in speed (Figure 1). These results demonstrate that RPM-1 plays a role in exploratory locomotion.

### The harsh touch response is impaired in *rpm-1* mutants

The defect in exploratory locomotion caused by loss *of rpm-1* function that we uncovered was relatively mild compared with the developmental defects described previously. Therefore, we postulated that more vigorous or complex behavior (requiring stronger or protracted activation of the nervous system) might be needed to detect more pronounced behavioral defects in *rpm-1* mutants. To test this, we examined a stimulus-evoked behavior, the harsh touch response.

**Figure 2:**
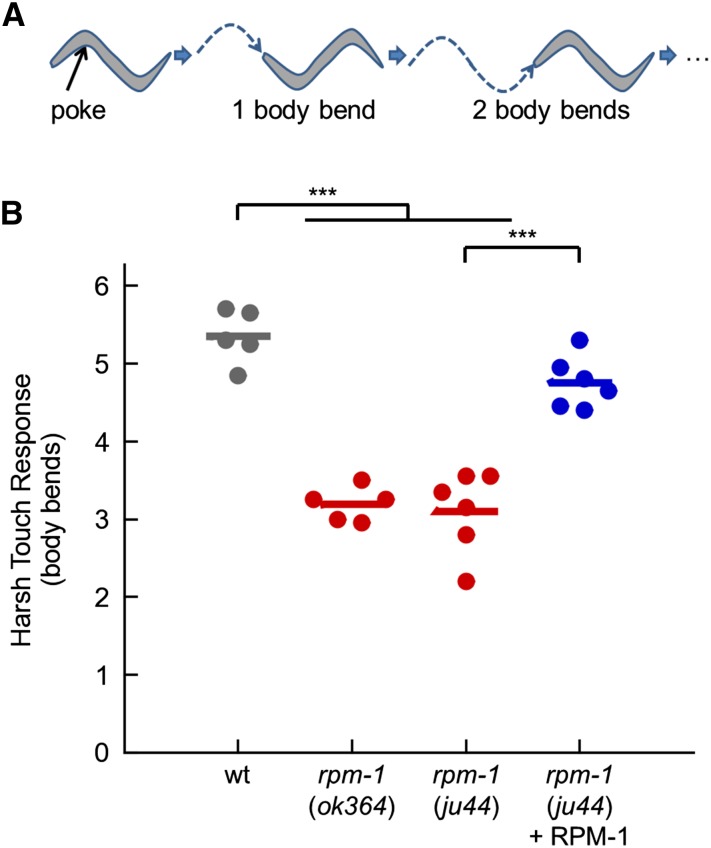
RPM-1 is required for the harsh touch response. (A) Schematic of *C. elegans* harsh touch response. (B) Harsh touch responses of wild-type animals (wt; gray), *rpm-1* (lf) mutants (red), and *rpm-1* mutants with a transgene that uses the native *rpm-1* promoter to express RPM-1 (blue). Response was measured as the number of reverse body bends an animal makes following a harsh touch stimulus to the head. Points represent the mean response of 20 animals on different experimental days. Lines represent the mean of all points within each genotype. For transgenic rescue (blue), each point represents an independently derived transgenic line. ***P < 0.001 for Student’s unpaired two-tailed *t*-test between indicated groups.

In *C. elegans*, harsh mechanical touch stimulates robust and extended reverse movement away from the stimulus (Chatzigeorgiou *et al.* 2010; Li *et al.* 2011; Way and Chalfie 1989). To quantitatively analyze locomotion following harsh touch, we counted the number of reverse body bends that occurred following a harsh stimulus to the anterior of the animal with a platinum wire (Figure 2A). In wild-type animals, harsh touch resulted in robust reverse movement (Figure 2B). In contrast, while all *rpm-1* mutants responded to the harsh touch stimulus (n = 300), we observed a 40% reduction in reverse locomotion after harsh touch (Figure 2B). This defect was rescued with transgenic extrachromosomal arrays that express RPM-1 using the native *rpm-1* promoter. These results show that RPM-1 is required for the response to harsh touch.

### Loss of RPM-1 affects habituation, a simple form of learning

Behaviors that require the nervous system to be plastic and change with experience, such as learning, rely on protracted and efficient neuron and circuit function, and therefore might reveal *rpm-1* defects. *C. elegans* is capable of a number of simple learning tasks (Ardiel and Rankin 2010; Sasakura and Mori 2013). We chose to test *rpm-1* mutants for defects in habituation, a form of learning in which animals learn to ignore repeated irrelevant stimuli.

**Figure 3:**
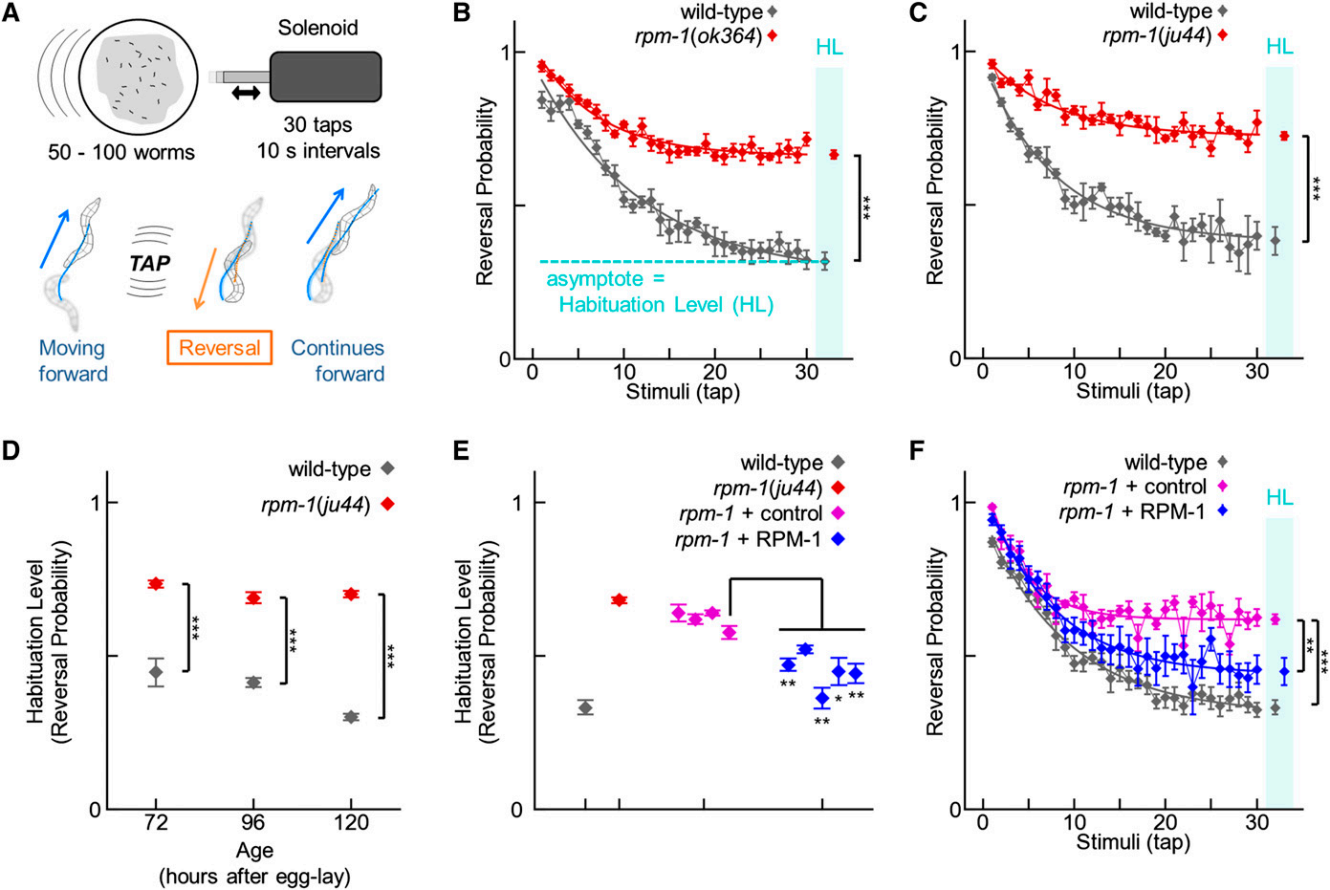
Loss of RPM-1 disrupts habituation, a simple form of learning. (A) Schematic of the tap stimulus and habituation protocol (top), and the reversal response that follows a tap stimulus (bottom). (B−C) Reversal probability after each tap stimulus of wild-type animals (gray) and *rpm-1* (lf) mutants (red). Connected points represent the mean ± SEM of the tap response for each stimulus (n = 3−6 experiments; each experiment consists of 50−100 animals). Smooth thick lines indicate the best-fit exponential curves. Habituation level was measured as the asymptote of the curve (plotted inside the cyan column labeled HL with ± SEM). (D) Habituation level (asymptote) of wild-type animals (gray) and *rpm-1* mutants at different ages. *rpm-1* mutants have a strong habituation defect at all ages tested. (E) Habituation level (asymptote) of wild-type animals (gray), *rpm-1* mutants (red), four independent transgenic lines of *rpm-1* mutants expressing transgenic co-injection markers (control; magenta), and five independent transgenic lines of *rpm-1* mutants in which the native *rpm-1* promoter is used to express RPM-1 (blue). All RPM-1−expressing lines were significantly lower than the median control line and 4/5 RPM-1 lines were significantly less than the minimum control line (asterisks). (F) Representative example of a complete tap habituation response profile for one of the transgenic lines summarized in panel E (median transgenic lines are shown). *P < 0.05; **P < 0.01; ***P < 0.001 for Student’s unpaired two-tailed *t*-test between indicated groups.

Previous work has shown that the tap stimulus, a brief knock against the side of a Petri plate that is a nonlocalized version of the gentle touch response, is ideal for assessing habituation (Giles and Rankin 2009; Rankin *et al.* 1990). The neural circuit that mediates the tap response has been characterized, and the neurons that form the circuit include the mechanosensory neurons, interneurons and motor neurons (Wicks and Rankin 1995). Previous work has shown that each of these types of neurons that make up the tap circuit have developmental defects in *rpm-1* mutants. Thus, tap habituation was a plausible behavioral readout to test for deficits in *rpm-1* mutants. Although tap activates both anterior and posterior mechanosensory neurons, which mediate reversal and acceleration respectively, the neural circuit that integrates tap input in adult animals is biased toward reversal (Wicks and Rankin 1995). As a result, 90% of naïve animals reverse in response to tap, and 10% accelerate or do not respond. An advantage of using the tap stimulus is that many worms can be stimulated at once, and the MWT can be used to record the behavior of a population of animals and detect the number of reversals in response to tap (Swierczek *et al.* 2011) (Figure 3A). Consistent with previous studies that examined gentle touch (Bounoutas *et al.* 2009; Schaefer *et al.* 2000), we found that *rpm-1* (lf) mutants did not have a defect in the initial response to tap stimulus. In fact, we found a very mild, but statistically significant, increase in the reversal probability in response to tap (Figure 3C, initial data point mean ± SEM: wild-type = 0.916 ± 0.008, n = 6; and *rpm-1*(*ju44*) = 0.960 ± 0.014, n = 4; *P* = 0.042). This effect was only detected in 7 of 15 replicate experiments that had a similar statistical design (*i.e.*, n = 3−6; data not shown). Analysis across replicates, where each n-value was the mean of one experiment, increased statistical power and provided further confidence that *rpm-1* mutants had a mild, but significant, increase in reversal probability to tap compared with wild-type animals (wild-type = 0.890 ± 0.008 and *rpm-1* (*ju44*) = 0.954 ± 0.003; n = 15 experiments with 3−6 trials per experiment; *P* < 10^−6^). In contrast, the magnitude of the tap response, measured by the duration of the response and the speed of the response, was not distinguishable from wild-type (duration mean ± SEM: wild-type = 2.74 ± 0.06 s and *rpm-1* (*ju44*) = 2.69 ± 0.03 s, n = 15, *P* = 0.41; speed mean ± SEM: wild-type = 0.282 ± 0.005 mm/s and *rpm-1*(*ju44*) = 0.279 ± 0.006 mm/s, n = 15, *P* = 0.88). These results show that *rpm-1* mutants have no deficits in registering or responding to a single tap stimulus.

When presented with repeated tap stimuli (30 taps with 10 sec interstimulus intervals), wild-type *C. elegans* habituate (Figure 3, A and B). This is detected as an exponential decrease in the probability of response to approximately 0.4 following multiple taps. We designated the habituation level as the asymptote of the best-fit curve for a population’s responses (Figure 3B). Both *rpm-1* (lf) mutants, *ok364* and *ju44*, had a significantly higher habituation level than wild-type animals (Figure 3, B and C). The failure of *rpm-1* mutants to decrease their response to tap over time indicated these animals had a defect in habituation.

The habituation level of wild-type animals decreases with age (Timbers *et al.* 2013). Therefore, a mutant that develops or ages slowly might appear to have a habituation defect if the animals are not staged carefully. We found that *rpm-1* mutants developed and aged at a similar rate to wild-type animals as assessed by the onset of the L4 larval stage and egg-laying in synchronized populations (data not shown). To confirm that habituation defects in *rpm-1* animals were not due to altered rates of aging, we tested wild-type animals and *rpm-1* mutants 24 hr before and after the age at which we normally assess habituation (96 hr). We found that *rpm-1* mutants had a significant defect in habituation at all ages tested (Figure 3D). This eliminates the possibility that habituation defects caused by *rpm-1* (lf) were due to delayed development or aging.

Next, we tested whether the lesions in *rpm-1* caused defects in habituation by performing transgenic rescues. *rpm-1* (lf) mutants were engineered with transgenic extrachromosomal arrays in which RPM-1 was expressed using the endogenous *rpm-1* promoter. Transgenic animals were generated using a *pha-1*(*e2123*) temperature-sensitive lethal mutant background to allow for rapid positive selection (Granato *et al.* 1994). This was a valuable approach when assessing hundreds of transgenic animals simultaneously using the MWT. Transgenic arrays also contained P_myo-2_mCherry, which was used to visually confirm transgenic animals. As a control, we created transgenic *rpm-1* mutants that had the *pha-1* mutation and transgenically expressed both PHA-1 and mCherry for selection, but lacked the *rpm-1* transgene. Transgenic expression of markers alone had a mild effect on the habituation defect in *rpm-1* mutants because three of four control lines had habituation levels slightly, but significantly, less than non-transgenic *rpm-1* mutants (Figure 3E, not indicated on graph; *P* < 0.05). However, we found that the RPM-1 expressing transgenic lines significantly rescued the habituation defect beyond the level of control lines (Figure 3, E and F). These results show that tap habituation is strongly impaired in animals lacking RPM-1.

### RPM-1 functions in the mechanosensory neurons to affect habituation

**Figure 4:**
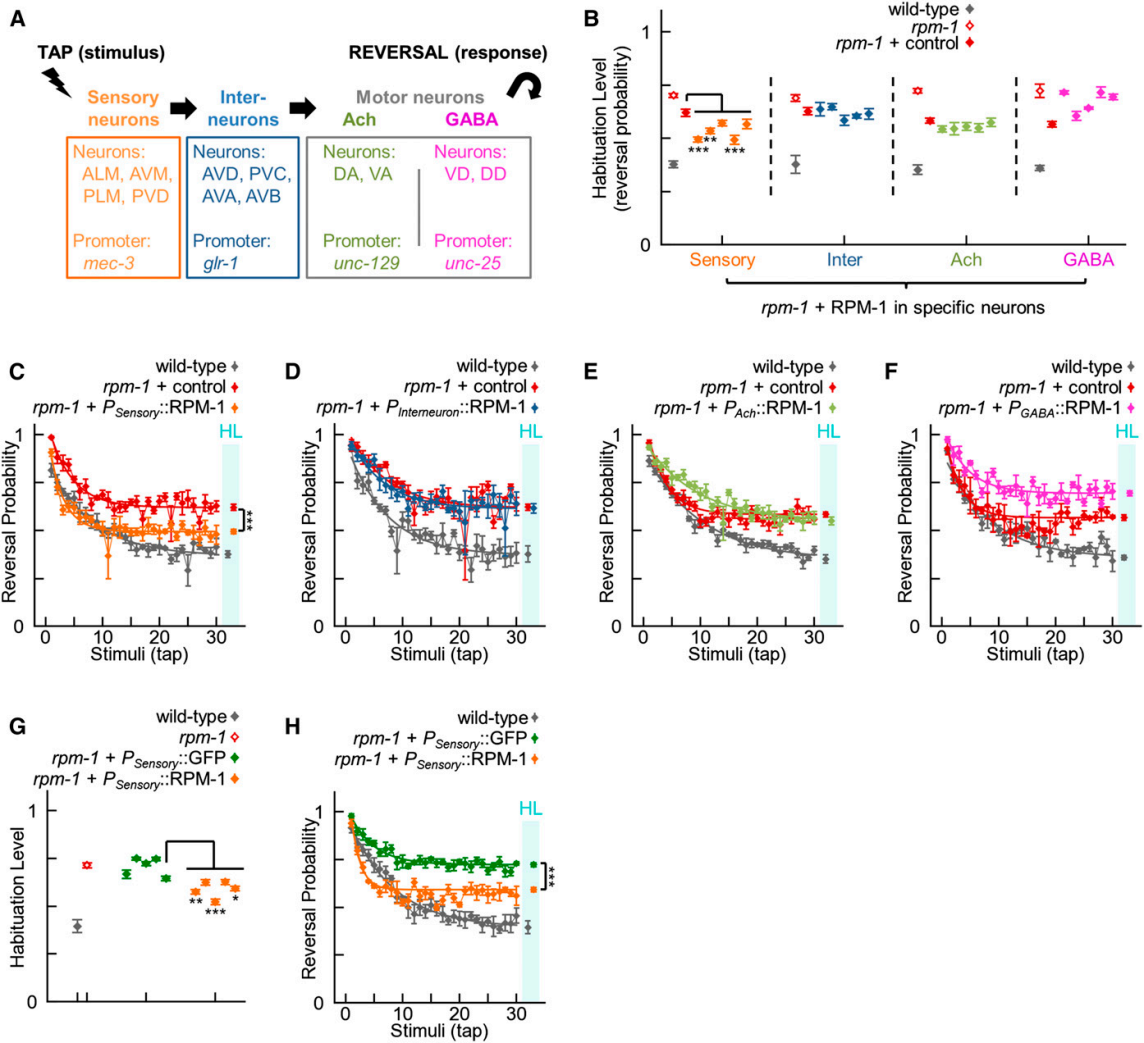
RPM-1 affects habituation to tap by functioning in the sensory neurons. (A) Summary of the neural circuit that mediates the tap withdrawal response, and promoters used to drive transgene expression in different parts of the circuit. (B) Habituation level of wild-type animals (gray), *rpm-1* (lf) mutants (open red), *rpm-1* mutants expressing transgenic coinjection markers (control; red), and five transgenic lines of *rpm-1* mutants in which RPM-1 is expressed in different neurons of the tap habituation circuit (various colors). Only expression of RPM-1 in the sensory neurons significantly rescued the mutant defect compared with the paired control line (3/5 lines, asterisks; orange *vs.* red). (C−F) Representative examples of complete tap habituation response profiles for the genotypes summarized in (B). Connected points represent the mean ± SEM of the tap response for each stimulus (n = 4−6 experiments). Smooth thick lines indicate the best-fit exponential curves. Points in the cyan bar labeled HL indicate the habituation level (asymptote of the curve ± SEM). Expression in the sensory neurons partially rescued the *rpm-1* mutant defect (C). (G) Habituation of wild-type animals (gray), *rpm-1* mutants (open red), and five transgenic lines of *rpm-1* mutants using the sensory neuron promoter to express GFP (green) or RPM-1 (orange). All transgenic lines expressing RPM-1 significantly rescued habituation defects compared with the median control line, and 3/5 RPM-1 lines were significantly rescued compared with the minimum control line (asterisks). Therefore, expression of RPM-1, and not the presence of the sensory neuron promoter in transgenic arrays, was responsible for rescuing habituation defects in *rpm-1* mutants. (H) Representative examples of complete tap habituation response profiles for the genotypes in panel G. For all panels, the *rpm-1* allele used was *ju44* and asterisks indicate transgenic lines that were significantly lower than the indicated control using Student’s unpaired two-tailed *t*-tests (*P < 0.05; **P < 0.01; ***P < 0.001). Note that different, independently derived transgenic lines were used for B and G, thus collectively 6/10 sensory neuron lines showed significant rescue.

The neural circuit that mediates the tap response in *C. elegans* has been well characterized (Wicks and Rankin 1995). It is composed of mechanosensory neurons, glutamate receptor-containing interneurons, and both cholinergic and GABAergic motor neurons (Figure 4A). RPM-1 is expressed in all the neurons in this circuit (Park *et al.* 2009; Schaefer *et al.* 2000; Zhen *et al.* 2000). To identify the portion of the tap circuit in which RPM-1 function affects habituation, we performed transgenic rescue experiments using neuron-specific promoters that restrict expression to a subset of neurons in the tap circuit (Figure 4A). We used the *mec-3* promoter for the sensory neurons (Way and Chalfie 1989), the *glr-1* promoter for the interneurons (Park *et al.* 2009), the *unc-129* promoter for the cholinergic motor neurons (Colavita *et al.* 1998), and the *unc-25* promoter for the GABAergic motor neurons (Jin *et al.* 1999). We tested five independently derived transgenic lines for each promoter. As a control, we analyzed *rpm-1*; *pha-1* animals that expressed the PHA-1 and mCherry transgenic selection markers but not RPM-1. Only expression of RPM-1 in the sensory neurons significantly rescued the habituation defect in *rpm-1* mutants (Figure 4, B−F). To control for possible effects caused by the *mec-3* promoter, we generated five independent transgenic lines in which *rpm-1* mutants expressed GFP using the *mec-3* promoter. The habituation levels in these *mec-3* promoter control lines were compared with five additional independently-derived transgenic lines that expressed RPM-1 using the *mec-3* promoter. As shown in Figure 4, G and H, RPM-1 expression significantly rescued the habituation defect compared with GFP expression. Taken together, our findings suggest that RPM-1 function in the mechanosensory neurons is required for normal tap habituation. However, our results do not definitively rule out the possibility that RPM-1 might function in other parts of the tap habituation circuit.

### RPM-1 is required throughout development for normal habituation

Although RPM-1 is an important player in neuronal development, RPM-1 continues to be expressed into adulthood, where it plays a role in axon regeneration(Hammarlund *et al.* 2009; Nix *et al.* 2011). Orthologs of RPM-1 in mouse and fly also function postdevelopmentally to regulate axon degeneration, axon regeneration, and aversive long-term memory (Babetto *et al.* 2013; Huang *et al.* 2012; Xiong *et al.* 2010, 2012). Although our initial hypothesis was that habituation defects in *rpm-1* mutants result from abnormalities in neuronal development, it is possible RPM-1 might function in adulthood to regulate habituation. To address this, we tested whether adult-specific expression of RPM-1 could rescue the habituation defect in *rpm-1* mutants.

To induce adult-specific expression of RPM-1, we used a heat shock promoter (P*hsp-16.2*) that allows temperature dependent induction of gene expression. P*hsp-16.2* has been used previously in a variety of contexts to temporally regulate gene expression in the *C. elegans* nervous system (Adler *et al.* 2006; Flames and Hobert 2009; Kurup *et al.* 2015; Stringham *et al.* 1992). Notably, P*hsp-16.2* was used to induce adult-specific expression in the mechanosensory neurons (where RPM-1 regulates habituation) and was previously used to assess the role of a gene involved in learning and memory (Ikeda *et al.* 2008; Xu *et al.* 2001). Thus, temporal control of RPM-1 expression using P*hsp-16.2* was a suitable way to determine whether RPM-1 regulates habituation by functioning in adulthood.

**Figure 5:**
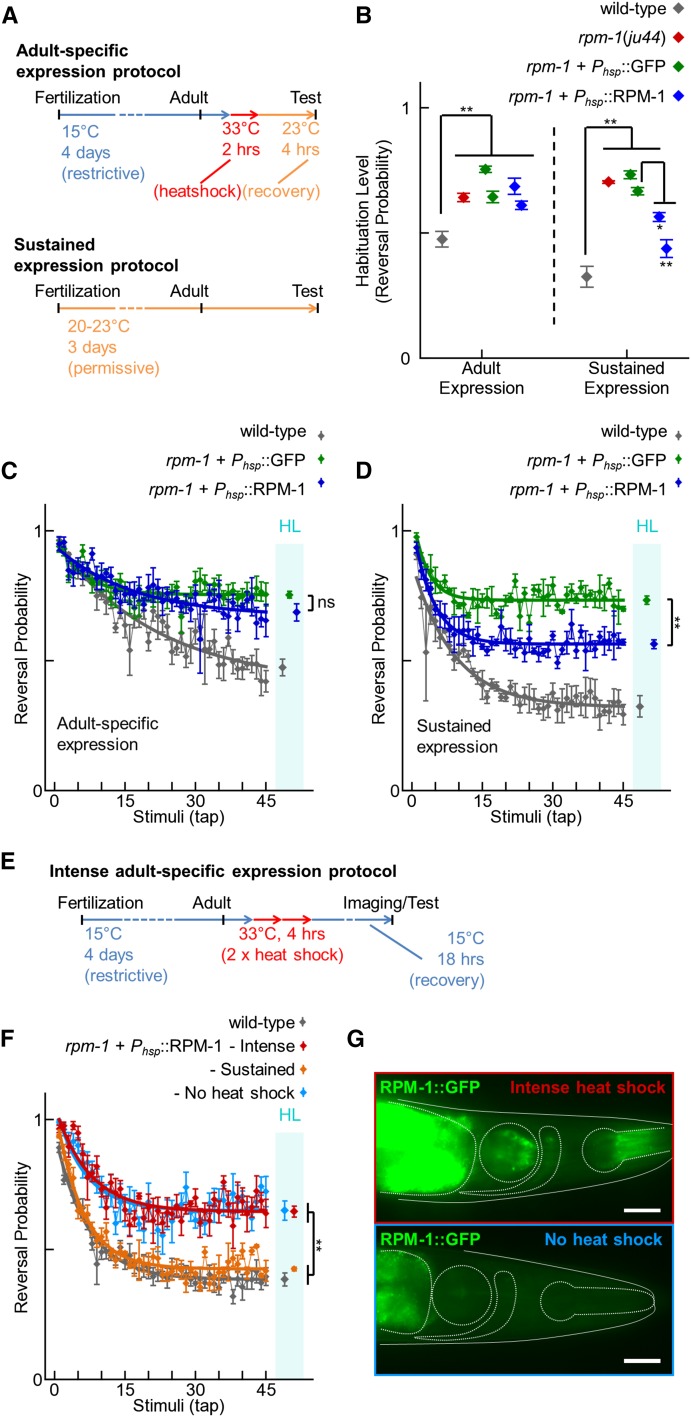
Adult-specific expression of RPM-1 does not rescue habituation defects in *rpm-1* mutants. (A) Schematic of the protocol for induction of adult-specific expression and sustained developmental expression of RPM-1 using the *hsp-16.2* heat shock promoter. (B) Habituation of wild-type animals (gray), *rpm-1* (lf) mutants (red), and *rpm-1* mutants expressing GFP (green) or RPM-1 (blue) using the heat shock promoter under the indicated expression conditions. Note that adult-specific expression of RPM-1 did not rescue the habituation defect in *rpm-1* mutants, whereas sustained expression of RPM-1 partially rescued the habituation defect in two independently derived transgenic lines compared with the minimum control line (asterisks). (C−D) Representative examples of complete tap habituation response profiles for the genotypes and conditions in panel B. Connected points represent the mean ± SEM of the tap response for each stimulus (n = 4−6 experiments). Smooth thick lines indicate the best-fit exponential curves. Points in the cyan bar labeled HL indicate the habituation level (asymptote of the curve ± SEM). (E) Schematic of the protocol for induction of intense adult-specific expression using the *hsp-16.2* heat shock promoter. (F) Habituation of wild-type animals (gray) and *rpm-1* mutants expressing RPM-1::GFP using the heat shock promoter under the indicated expression conditions (various colors). Data are represented as in (C) and (D). (G) Images of the head (solid outline) of P*hsp-16.2*::RPM-1::GFP positive *C. elegans* 18 hr after heat shock at 33° for 4 hr (top panel) or not heat shocked (bottom panel). Four internal structures are dash-outlined (from left to right): anterior gut (incomplete shape), posterior pharynx (circle), nerve ring (comma-shaped), and anterior pharynx (extended circle). Only heat shocked animals had visible RPM-1::GFP expression in the nerve ring. Stronger RPM-1::GFP fluorescence was present in the gut and pharynx. Low level autofluorescence is present in gut without heat shock (bottom panel). Scale bar is 25 μm. ns = not significant, *P < 0.05, **P < 0.01 for Student’s unpaired two-tailed *t*-tests between indicated groups.

For adult-specific expression, we cultivated transgenic animals at 15° (a restrictive temperature for P*hsp-16.2*) until the young adult stage and then shifted to 33° (a strongly permissive temperature) for 2 hr (Figure 5A). The studies mentioned above successfully induced P*hsp-16.2* with various durations of heat shock ranging from 15 min to 4 hr. We chose 2 hr because it was the most commonly used duration and was used previously to rescue a learning phenotype (Ikeda *et al.* 2008). After heat shock, animals were allowed to recover for 4 hr at 20−23° and were then tested for habituation. *rpm-1* (lf) mutants were engineered with extrachromosomal arrays that used P*hsp-16.2* to express RPM-1 or GFP (negative control). *rpm-1* mutants that expressed RPM-1 in adulthood did not rescue habituation defects compared with mutants expressing GFP (Figure 5, B and C). In contrast, when transgenic animals were cultivated at 20−23° (a mildly permissive temperature for P*hsp-16.2*) for their entire lifespan prior to testing, we found that animals expressing heat shock promoter driven RPM-1 had reduced defects in habituation compared with animals that expressed GFP (Figure 5, B and D). Wild-type animals habituated to tap differently when cultivated at 15° *vs.* 23° (Figure 5, B−D). The habituation level was slightly greater at 15° and the habituation rate (the half-life of the exponential curve) was longer at 15°. This could be the result of slight differences in aging that occur with different cultivation temperatures as habituation is sensitive to age (Timbers *et al.* 2013). Although we attempted to age match animals from different cultivation temperatures based on the well characterized timing of developmental and aging landmarks at various cultivation temperatures (Byerly *et al.* 1976), slight differences in age could still have occurred. Alternatively, the heat shock promoter could affect endogenous heat shock protein levels and modestly impair habituation. To ensure differences were not simply a failure to reach maximal habituation levels, we habituated animals to 45 stimuli instead of 30. We also confirmed that statistical power was sufficient for adult temperature shift experiments to detect a rescue proportional to the rescue observed with cultivation at 23°.

Our results suggest that adult-specific expression of RPM-1 is not sufficient to rescue the habituation defects in *rpm-1* mutants. However, it was possible sufficient RPM-1::GFP expression was not occurring with our heat shock induction protocol. Therefore, we induced RPM-1::GFP expression in the stronger of our two rescuing lines with a more intense heat shock protocol (4 hr total: two sessions at 33° for 2 hr separated by a 30-min rest; Figure 5E) (Stringham *et al.* 1992). Even with intense heat shock induction of RPM-1::GFP, we observed no rescue of habituation defects in *rpm-1* mutants (Figure 5F). Expression of RPM-1::GFP was detected in the nervous system after intense heat shock (Figure 5G). Therefore, it is unlikely that adult-specific expression of RPM-1 failed to rescue habituation defects in *rpm-1* mutants because of technical issues with heat shock induced expression. Instead, it is likely that the tap habituation defect in *rpm-1* mutants is a consequence of the developmental abnormalities in these animals.

### Molecules that mediate RPM-1 function in development also affect habituation

**Figure 6:**
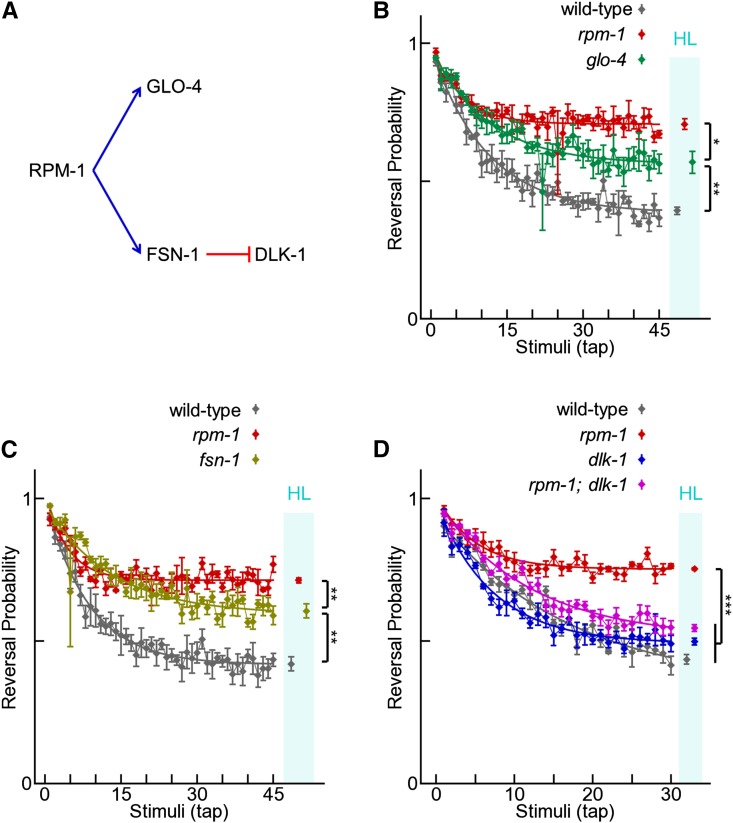
Molecules that mediate RPM-1 function in neuronal development affect habituation. (A) Known mediators of RPM-1 function in development; blue arrows represent activation and red bars represent inhibition. (B−D) Tap habituation response profiles for the indicated genotypes. Connected points are means ± SEM of tap response (n = 4−6 experiments). Points in cyan bar are habituation levels (HL; asymptotes of fitted curves ± SEM). Note that *glo-4* (B) and *fsn-1* (C) mutants caused a strong, but partial, habituation defect compared with wild-type and *rpm-1* mutants. *dlk-1* (lf) strongly suppressed the habituation defect caused by *rpm-1* (lf), as indicated by habituation levels in *rpm-1*; *dlk-1* double mutants compared with *rpm-1* single mutants (D). *P < 0.05; **P < 0.01; ***P < 0.001 for Student’s unpaired two-tailed *t*-tests between indicated groups.

Several downstream proteins that mediate the function of RPM-1 in neuronal development have been identified (Baker *et al.* 2014, 2015; Grill *et al.* 2007, 2012; Liao *et al.* 2004; Nakata *et al.* 2005; Tulgren *et al.* 2014; Yan *et al.* 2009). For example, RPM-1 positively activates GLO-4 to regulate the GLO-1 Rab GTPase (Figure 6A) (Grill *et al.* 2007). RPM-1 also functions in a complex with the F-box protein, FSN-1, to negatively regulate the DLK-1 MAP3K (Figure 6A) (Grill *et al.* 2007; Liao *et al.* 2004; Nakata *et al.* 2005). If the developmental abnormalities in *rpm-1* mutants result in habituation defects, we would expect habituation to also be affected by molecules that mediate RPM-1 function in development.

RPM-1 function is mediated in part by GLO-4 and FSN-1 (Grill *et al.* 2007). As a result, animals that have a loss-of-function mutation in either *glo-4* or *fsn-1* have mild developmental defects compared with *rpm-1* mutants. We tested habituation of *glo-4* (*ok623*) and *fsn-1* (*hp1*) and found that *glo-4* mutants and *fsn-1* mutants each had significantly higher habituation levels than wild-type animals, but lower levels than *rpm-1* mutants (Figure 6, B and C). These results are consistent with GLO-4 and FSN-1 mediating RPM-1 function in habituation.

*dlk-1* (lf) mutations strongly, but partially, suppress developmental defects in *rpm-1* mutants, such as impaired axon termination and synapse formation (Grill *et al.* 2007; Nakata *et al.* 2005). We tested whether this genetic relationship also existed for habituation. *dlk-1* (*tm4024*) mutants had similar habituation to wild-type animals, and *rpm-1*; *dlk-1* double mutants had suppressed habituation defects compared with *rpm-1* single mutants (Figure 6D). Thus, *dlk-1* and *rpm-1* show similar genetic relationships in the context of both habituation and neuronal development. These findings support the proposition that habituation defects in *rpm-1* mutants arise from defects in neuronal development.

## Discussion

After more than a decade of research, the PHR proteins have emerged as key, conserved regulators of neuronal development and axon degeneration (Conforti *et al.* 2014; Po *et al.* 2010). Numerous downstream effector proteins and pathways that mediate PHR protein function have also been discovered (Baker *et al.* 2014, 2015; Collins *et al.* 2006; Grill *et al.* 2007, 2012; Liao *et al.* 2004; Murthy *et al.* 2004; Nakata *et al.* 2005; Tian *et al.* 2011; Tulgren *et al.* 2014; Xiong *et al.* 2012; Yan *et al.* 2009). Despite this important progress, a lingering question has remained: how does the function of PHR proteins during development impact behavior? To address this question, we used a broad, quantitative approach by testing three diverse behaviors mediated by neurons known to express RPM-1: spontaneous exploratory locomotion, evoked harsh touch response, and tap habituation (a simple but critical form of short-term learning).

Using the MWT to quantitatively analyze exploratory locomotion, we observed defects in *rpm-1* (lf) mutants (Figure 1). *rpm-1* mutants spend slightly more time performing reversals and their speed is 15–20% slower than wild-type animals. Previous studies in *Drosophila* hinted at this possibility, as qualitative analysis of *Highwire* mutants described a mild walking defect (Wan *et al.* 2000) and *rpm-1* mutants were previously shown to trend toward decreased locomotion (Noma *et al.* 2014). Our observation, combined with previous findings, suggests that loss of PHR protein function in invertebrates leads to relatively mild defects in locomotion. The role of PHR proteins in vertebrate locomotion has not been explored due to early larval lethality in fish (Odenthal *et al.* 1996), and death shortly after birth in mice lacking Phr1 (Burgess *et al.* 2004; Lewcock *et al.* 2007; Bloom *et al.* 2007). However, the motor neurons of Phr1 mutant and knockout mice have axon extension and synapse formation defects, which suggests that locomotion is likely to be impaired if Phr1 were specifically ablated in these neurons.

Given that *rpm-1* mutants have abnormalities in synapse formation, but not complete loss of synaptic connections (Schaefer *et al.* 2000; Zhen *et al.* 2000), we postulated that behaviors requiring intense, protracted, or complex activity of the nervous system might be more sensitive to *rpm-1* dysfunction. Consistent with this idea, *rpm-1* mutants had relatively severe defects in their response to harsh touch, which requires coordinated and protracted circuit function (Figure 2). To expand on this idea, we investigated how *rpm-1* mutants habituate to tap, a form of short-term learning that takes place over several minutes of repeated stimulation. We found that *rpm-1* mutants had slight hypersensitivity to the initial tap stimulus, but extremely strong defects in habituation to tap (Figure 3). Overall, our results suggest that the more protracted or complex a given behavior, the more it will be impaired with loss of *rpm-1* function.

Habituation defects in *rpm-1* mutants were particularly pronounced. To our knowledge, *rpm-1* mutants have some of the strongest defects in habituation detected to date. Importantly, a converging series of observations supported our finding that RPM-1 affects habituation including: multiple alleles of *rpm-1* having habituation defects (Figure 3), rescue of habituation defects by transgenic expression of RPM-1 (Figure 3), and known mediators of RPM-1 signaling (*i.e.*, GLO-4 and FSN-1) affecting habituation (Figure 6). Transgenic rescue using drivers that are specific for subsets of neurons in the tap circuit showed that RPM-1 affects habituation by functioning in the mechanosensory neurons (Figure 4). It should be noted that because we only performed rescues with single drivers at a time, our results do not rule out the possibility that RPM-1 also functions in a combination of other neuron types in the tap circuit. *rpm-1* mutants also have synapse formation defects in the chemosensory neurons (Crump *et al.* 2001), which mediate simple forms of behavioral plasticity such as adaptation (Colbert and Bargmann 1995), habituation (Wen *et al.* 1997), and associative learning (Saeki *et al.* 2001; Wen *et al.* 1997). Thus, the effect of RPM-1 on learning might generalize beyond the tap response circuit to chemosensory responses.

Several of our findings support the conclusion that impaired habituation in *rpm-1* mutants is likely to be the consequence of developmental defects in these animals. First, adult-specific expression of RPM-1 was unable to rescue habituation defects in *rpm-1* (lf) mutants, whereas expression of RPM-1 throughout development rescued habituation defects (Figure 5). Second, mediators of RPM-1 function in development (*i.e.*, GLO-4, FSN-1, and DLK-1) also affected habituation (Figure 6). Thus, our findings suggest that RPM-1 function in neuronal development is critical for normal habituation. In contrast to our results, a previous study found that Highwire, the *Drosophila* ortholog of RPM-1, functions in adulthood to properly gate long-term aversive memory (Huang *et al.* 2012). Taken together, these observations suggest two distinct roles for PHR proteins in learning and memory: 1) a role in the development of neurons, which has a critical consequence on short-term learning later in adulthood, and 2) a role in regulating the consolidation of long-term memories specifically in adulthood.

How might disrupted neuron development in *rpm-1* (lf) mutants lead to defects in habituation? *rpm-1* mutants have two developmental defects in their mechanosensory neurons, abnormal synapse formation and overextended axons as a result of failed axon termination (Grill *et al.* 2007; Schaefer *et al.* 2000). The chemical synapses of the mechanosensory neurons are not critical for the tap response itself, which is likely mediated by electrical synapses (Chalfie *et al.* 1985; Wicks and Rankin 1995). This finding is consistent with our observation that *rpm-1* mutants had intact tap responses. However, one cellular mechanism of habituation is chemical synapse plasticity (Bailey and Chen 1988; Castellucci *et al.* 1970; Castellucci and Kandel 1974; Das *et al.* 2011; Davis *et al.* 1982; Gover *et al.* 2002; Krasne 1969; Lingenhohl and Friauf 1994; Rankin and Wicks 2000; Simons-Weidenmaier *et al.* 2006). Therefore, it is possible that impaired formation of chemical synapses in *rpm-1* mutants might explain the defects in habituation we have uncovered in these animals. Another cellular mechanism of habituation is the modulation of neuron excitability (Kindt *et al.* 2007). Mechanosensory axons contain voltage-gated calcium channels that propagate the signal from sensory transduction (Frokjaer-Jensen *et al.* 2006; Goodman 2006; O’Hagan and Chalfie 2006; Suzuki *et al.* 2003). Therefore, it is possible that the abnormally long mechanosensory axons in *rpm-1* mutants might alter neuron excitability thereby disrupting habituation. At this point, it remains difficult to determine which of these possibilities is more likely. It is also possible the dramatic habituation defects in *rpm-1* mutants could stem from a combination of both developmental abnormalities.

Abnormalities in short-term learning are often associated with neurodevelopmental disorders. Specifically, habituation defects have been observed in patients with schizophrenia (Akdag *et al.* 2003; Bolino *et al.* 1992; Braff *et al.* 1992; Ludewig *et al.* 2003; Meincke *et al.* 2004; Parwani *et al.* 2000; Taiminen *et al.* 2000), autism (Green *et al.* 2015; Guiraud *et al.* 2011; Kleinhans *et al.* 2009; Lombardo *et al.* 2009), and fragile X syndrome (Bruno *et al.* 2014; Van der Molen *et al.* 2012). Several molecules in PHR protein signaling pathways also are associated with schizophrenia and autism, including the Tuberous Sclerosis Complex (Han *et al.* 2012; Murthy *et al.* 2004), FSN-1/Fbxo45 (Sagar *et al.* 2013; Wang *et al.* 2014), and ESS-2/DGCR14 (Noma *et al.* 2014). Our finding that RPM-1 and FSN-1 affect habituation, most likely through roles in neuronal development, provides behavioral evidence from a model system to support the growing genetic associations between PHR protein signaling and neurodevelopmental disorders.
